# Biographical Sketch of Dental Pathology and Practice

**Published:** 1896-05

**Authors:** Frank Abbott


BIOGRAPHICAL SKETCH OF DENTAL PATHOLOGY AND PRACTICE. By Frank Abbott, M.D.

This is a work of 240 pages recently issued by the S. S. White Manufacturing Company. It consists of twenty-two chapters, and presents the views of the author very clearly and concisely on the histology of the teeth, but more especially with reference to the pathology of enamel and dentine, with some special reference to the etiology of caries. He also embraces microscopic absorption of temporary teeth ; a chapter on exposed pulps and their treatment, and the management of pulpless teeth and alveolar abcess. He also devotes a chapter to the diseases of the antrum, especially with reference to dental complications and their treatment, salivary calculus and pyorrhœa alveolaris, also facial neuralgia, hypertria of cement, etc.

Dr. Abbott has formed a very valuable work for the profession in bringing together in this form his views upon these and other topics which have from time to time been presented before the American Dental Association and elsewhere. Much additional matter, however, is embraced in this volume, and as a whole it is a very valuable work. While, of course, there are points presented upon some of these subjects about which there is a diversity of opinion in the profession, and some of which will afford ground for criticism by some, it has perhaps as few objectionable features as any work of the kind would be likely to have. It will be a very valuable work for both the student and the practitioner. The defective or abnormal features of the enamel is, perhaps, as fully and clearly set forth in this work as will be found anywhere else. This is a subject to which Dr. Abbott has given a large share of attention under the advantages of superior opportunity and aid. Dr. Abbott is entitled to great credit for the production of this work, and, we doubt not, it will in a large measure be accorded him.

